# SE: an algorithm for deriving sequence alignment from a pair of superimposed structures

**DOI:** 10.1186/1471-2105-10-S1-S4

**Published:** 2009-01-30

**Authors:** Chin-Hsien Tai, James J Vincent, Changhoon Kim, Byungkook Lee

**Affiliations:** 1 Molecular Modeling and Bioinformatics Section, Laboratory of Molecular Biology, Center for Cancer Research, National Cancer Institute, National Institutes of Health, Bethesda, MD 20892, USA; 2 Bioinformatics Core, Vermont Genetics Network, Department of Biology, University of Vermont, Burlington, VT 05405, USA

## Abstract

**Background:**

Generating sequence alignments from superimposed structures is an important part of many structure comparison programs. The accuracy of the alignment affects structure recognition, classification and possibly function prediction. Many programs use a dynamic programming algorithm to generate the sequence alignment from superimposed structures. However, this procedure requires using a gap penalty and, depending on the value of the penalty used, can introduce spurious gaps and misalignments. Here we present a new algorithm, Seed Extension (SE), for generating the sequence alignment from a pair of superimposed structures. The SE algorithm first finds "seeds", which are the pairs of residues, one from each structure, that meet certain stringent criteria for being structurally equivalent. Three consecutive seeds form a seed segment, which is extended along the diagonal of the alignment matrix in both directions. Distance and the amino acid type similarity between the residues are used to resolve conflicts that arise during extension of more than one diagonal. The manually curated alignments in the Conserved Domain Database were used as the standard to assess the quality of the sequence alignments.

**Results:**

SE gave an average accuracy of 95.9% over 582 pairs of superimposed proteins tested, while CHIMERA, LSQMAN, and DP extracted from SHEBA, which all use a dynamic programming algorithm, yielded 89.9%, 90.2% and 91.0%, respectively. For pairs of proteins with low sequence or structural similarity, SE produced alignments up to 18% more accurate on average than the next best scoring program. Improvement was most pronounced when the two superimposed structures contained equivalent helices or beta-strands that crossed at an angle. When the SE algorithm was implemented in SHEBA to replace the dynamic programming routine, the alignment accuracy improved by 10% on average for structure pairs with RMSD between 2 and 4 Å. SE also used considerably less CPU time than DP.

**Conclusion:**

The Seed Extension algorithm is fast and, without using a gap penalty, produces more accurate sequence alignments from superimposed structures than three other programs tested that use dynamic programming algorithm.

## Background

Structure comparison and accurate structure-based sequence alignment are essential operations in structural bioinformatics. As of September 2008, the total number of structures in the Protein Data Bank (PDB) [1,2] is more than 53000 and is increasing by 20% per year. Good structure comparison algorithms are necessary in order to compare and classify these structures and to derive accurate sequence alignments, which can help establish evolutionary relationships among the proteins.

Many protein structure alignment programs include iterations of a two-step cycle: first superposing the two structures according to a given sequence alignment, and then deriving a new sequence alignment from the superimposed structures. Dynamic programming algorithm [3,4] is a widely used method for the second step. Programs such as SSAP [5], STRUCTAL [6], LSQMAN [7], CE [8], MATRAS [9], SHEBA [10], FAST [11] and others [12] use it to generate the alignments. However, dynamic programming algorithm requires using a gap penalty function, for which there is little guidance. It also uses a score function that usually considers only the distance between matching residues. Use of such a function can introduce incorrect alignments.

In order to recognize residue pairs that are structurally equivalent but not necessarily the closest ones and to avoid using a gap penalty function, we devised a novel algorithm called Seed Extension (SE) for obtaining the sequence alignment from a pair of superimposed structures. The performance of the new algorithm was compared with those of three programs that use the dynamic programming algorithm, namely LSQMAN, CHIMERA [13] and DP, which is a program extracted from SHEBA. LSQMAN and CHIMERA are two well-known programs and were chosen because they were easily available and could, without any modification, accept two superimposed structures and output the sequence alignment. The manually curated alignments in the Conserved Domain Database (CDD) [14] were used as the gold standard. Our results show that SE is fast and generates more accurate alignments, especially in cases where sequence or structural similarity is low. The program can be incorporated into an existing structure comparison program or it can simply be appended to such a program to improve its alignment quality.

## Results

### SE improves the accuracy of sequence alignments

**Figure 1:**
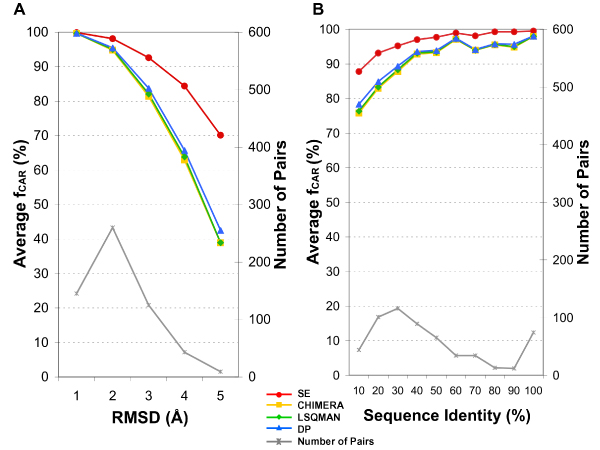
**Comparison of the average alignment accuracy**. The 582 superimposed pairs of proteins were binned according to (A) the RMSD or (B) the sequence identity of CDD alignment and the average fraction of correctly aligned residues (*f*_*CAR*_) were averaged over each bin for each program. The X-axis gives the bins labeled by the upper limit of the range. The numbers of structure pairs in each bin are shown in grey using the right-side Y-axis scale.

Figures 1A and 1B show the average fraction of correctly aligned residues, *f*_*CAR*_, in each RMSD and sequence identity ranges respectively by different programs. As expected, both SE and dynamic programming algorithms generated correct sequence alignments for structurally similar pairs. However, for pairs with RMSD greater than 2 Å, the average *f*_*CAR *_of SE was 9% to 28% better than those of programs using dynamic programming algorithm. For pairs with less than 40% sequence identity, the improvement was 3% to 8%. While CHIMERA, LSQMAN and DP yielded the average accuracy of 89.9%, 90.2% and 91.0% respectively over the 582 pairs of superimposed proteins, SE gave an average *f*_*CAR *_of 95.9%.

**Figure 2:**
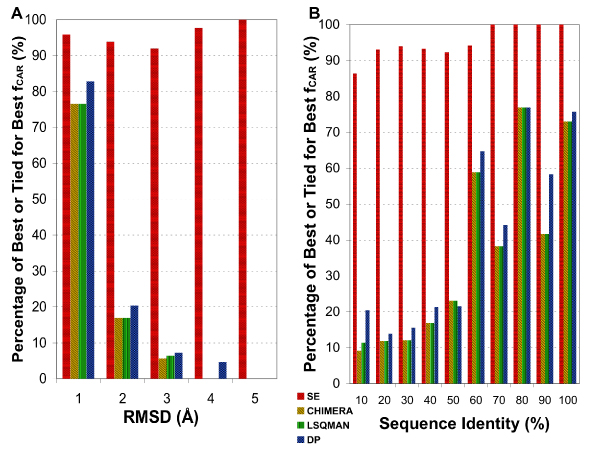
**Frequency with which a program generated the best *f*_*CAR *_or tied for the best**. The X-axis gives the RMSD (A) or sequence identity (B) bins as in Figure 1. The Y-axis gives the percentage of structure pairs within each bin for which a given program performed best or tied for the best.

We also compared the frequency that a program gave best alignment for each pair. The fraction of pairs for which a given program generated the highest *f*_*CAR*_, or tied with another programs with the highest *f*_*CAR*_, in different RMSD and sequence identity ranges are shown in Figures 2A and 2B, respectively. It is clear that SE generated almost always (never less than 85% of the time) the best alignment in all RMSD and homology ranges. The superior performance generally becomes more pronounced as the RMSD increases or the sequence identity decreases. Overall, SE generated the best alignment for 94.3% of the pairs if ties are included and 66.7% if ties are not counted.

**Figure 3:**
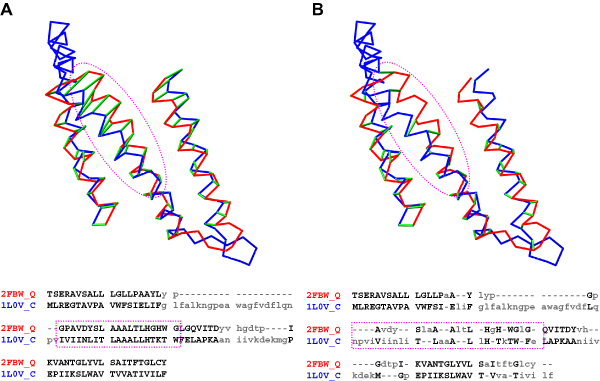
**Alignments of a sample pair**. The two structures in CD03493 , 2FBW_Q (red) and 1L0V_C (blue) were superimposed according to the CDD alignment in both panels A and B. The RMSD is 3.88 Å and the sequence identity is 8%. Pseudobonds in green indicate the aligned pairs from SE (Panel A) and DP (panel B). The sequence alignment is shown below in both panels; residues in bold upper case letters are aligned, others are not. The region in the magenta dashed squares corresponds to the magenta dashed oval in the superimposed structures above.

Figure 3 shows an example of the structure superposition and sequence alignment of a 3-helical bundle structure pair in the cd03439 family. The structures were superimposed according to the CDD alignment. The aligned pairs generated from different programs are indicated in green pseudobonds in the superimposed structures and in bold characters in the sequence alignments. Panel A shows the alignment from SE, which agrees 100% with CDD. The green pseudobonds indicate three well-aligned regions, which are also evident in the sequence alignment. On the other hand, panel B shows the alignment from DP which indicates only two well-aligned regions; the third region, enveloped in magenta dotted line, is poorly aligned with many gaps.

### SHEBA also generates more accurate alignment when the dynamic programming algorithm is replaced by SE

In order to see if the SE algorithm improves the alignment quality of structure comparison programs, it was implemented in SHEBA to replace the original dynamic programming algorithm. In SHEBA, after the initial alignment is found for a given pair of structures, they are superimposed according to Kabsch [15,16] and then a new sequence alignment is obtained from the superimposed structures using either the original DP routine or the new SE procedure. This Kabsch-DP or Kabsch-SE refinement cycle is repeated until convergence or until a set number of cycles has been completed.

**Figure 4:**
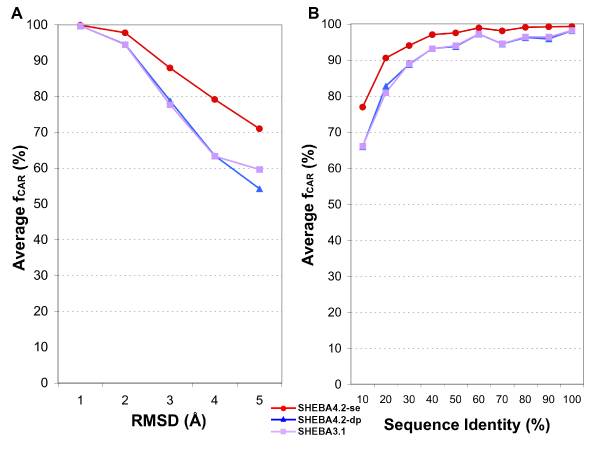
**Comparison of average alignment accuracy between SE and DP in SHEBA**. SE was implemented in SHEBA4.2 and compared with the DP routine of the new iteration scheme, SHEBA4.2-DP, and with the original scheme SHEBA3.1 (see Methods). The *f*_*CAR *_values were averaged over different RMSD (A) and sequence identity (B) bins as in Figure 1.

The average *f*_*CAR *_values obtained by the new version SHEBA4.2 with the DP or the SE routines are shown in Figure 4. Also included are the results from the original version, SHEBA3.1, which uses only the DP and a slightly different iteration scheme (see Methods). As can be seen, SHEBA with Kabsch-SE cycle generated more accurate alignments than the versions with the dynamic programming algorithm, regardless of the iteration scheme. On average, the *f*_*CAR *_of SHEBA with SE was 5% better than SHEBA with DP. For pairs with RMSD larger than 2 Å, SHEBA with SE was 9% to 12% better while for those with sequence identity less than 40%, the improvement was 4% to 10%.

### Execution time comparison

**Figure 5:**
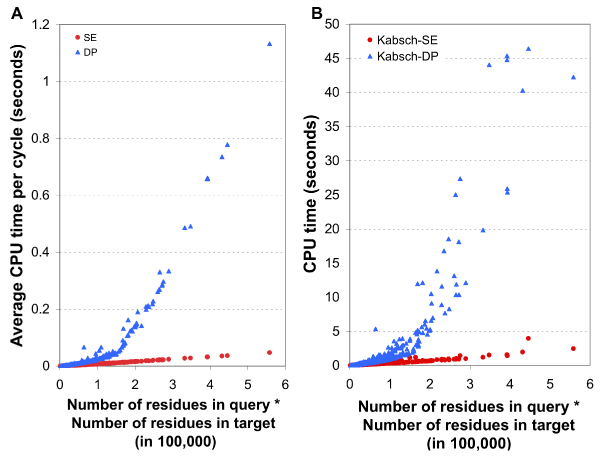
**Comparison of CPU times of SE and DP in SHEBA**. The CPU time to execute the SE and DP routine in SHEBA are plotted for each structure pair against the product of the lengths of the two proteins. (A) Average CPU time to execute one cycle of SE or DP. (B) CPU time to complete the Kabsch-SE and Kabsch-DP procedure, which involves multiple cycles of SE or DP and the Kabsch superposition process.

To compare the speed of SE and DP algorithms, we measured the CPU time spent on SE and DP routines implemented in SHEBA. In Figure 5, panel A shows the average CPU time per cycle of SE (red dots) and DP (blue dots) routines as a function of the product of the sizes of the two proteins compared for the 582 structurally similar pairs. The SE time increases nearly linearly with the product of the sizes whereas the DP time clearly increases much faster. A similar trend is seen in panel B, which gives the total CPU time taken for complete iteration of the refinement cycles for each pair of structures. It should be noted that SHEBA with DP and SHEBA with SE may run different numbers of cycles for a given pair of structures, depending on when the alignment converges. But the average number of cycles was comparable, 41 for SE and 49 for DP. SHEBA with SE was two times faster, on average, than SHEBA with DP, for proteins with 200 residues and more than 10 times faster for some larger protein pairs.

**Figure 6:**
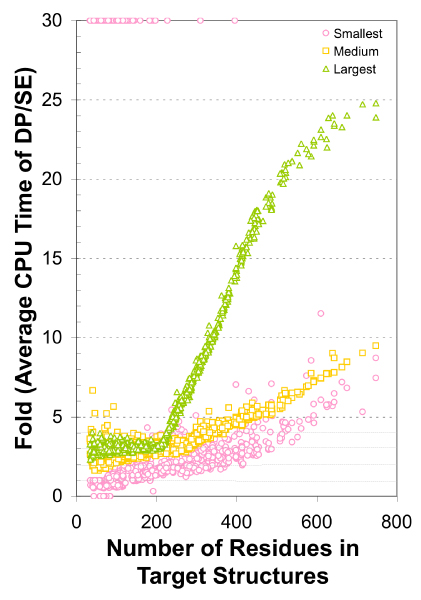
**Comparison of CPU times of SE and DP in SHEBA for one against all structure comparisons**. Three proteins were selected, the smallest (1RYT , 33 residues, in magenta), a medium sized (3AKY , 204, orange) and the largest (1H18_A , 747, green). Structure comparisons were made between each of these and all other 1164 domains using SHEBA with SE or DP. The average CPU time executed per DP cycle was divided by that per SE cycle for each pair and the ratio was plotted against the size of the target protein.

Above timing was for comparing a pair of similar structures. However, a common use of a structure comparison program is to search for similar structures in a structure database, which typically contains many more dissimilar structures than similar ones. In order to see how fast SE algorithm runs for dissimilar structures, we selected three CDD domains, 1RYT ,3AKY  and 1H18_A , with the smallest (33 residues), medium (204 residues) and the largest (747 residues) number of residues and compared each to all 1164 CDD domains with PDB structure. The CPU time used by DP routine divided by the CPU time used by SE for each pair is plotted against all the 1164 target structures in Figure 6. For the medium size query, SE is more than two times faster than DP when the target structure has 200 residues and more than 5 times faster when the target has 500 or more residues.

## Discussion

Obtaining the best sequence alignment from a pair of superimposed structures is a non-trivial problem when the two structures are not entirely similar. The common practice is to select a maximal number of aligned residue pairs that will minimize the aggregate sum of distances between Cα atoms of the selected pairs. The natural algorithm for doing this is the dynamic programming algorithm.

However, blind minimization of the distance sum, in conjunction with the use of an essentially arbitrary gap penalty function, can produce poor alignments. The problem is particularly easy to see when two structurally equivalent helices cross each other at an angle as in the case shown in Figure 3. In such cases, insufficient gap penalty often leads to an alignment of the closest, but not necessarily structurally equivalent, residues, with many gaps.

The SE algorithm is a heuristic algorithm, which approximately follows the mental process that one of the authors (BL) goes through when he manually writes down the alignment from visual inspection of a pair of superimposed structures displayed on a computer screen. It starts with a few residue pairs that are clearly equivalent and then extends the alignment without introducing a gap until the inter-residue distance changes abruptly. There is no explicit notion of a gap penalty, although it is implicitly present since the algorithm attempts to extend the alignment without a gap. We have shown in this study that this algorithm produces more accurate alignments than the dynamic programming algorithms implemented in three different programs. It is also considerably faster than the latter, especially when the structures are large. An additional merit of the algorithm is that it generates strictly symmetric alignments, i.e. it produces the same alignment when the query and target structures are swapped. This is not always the case with the dynamic programming algorithm.

The algorithm requires several parameters, including the distance change cutoff value, which is used to decide when to stop extension of the alignment, the scalar product threshold value, which measures the similarity of orientation of residue triplets and which is used to identify the seed alignments, and the distance tolerance and the sequence similarity cutoff values, which are used to decide when to consider the sequence similarity in choosing among a couple of conflicting alignments. Initially, we chose the values of these parameters intuitively. The values of the first two parameters were then varied within a limited range and the optimal values were chosen using the 582 pairs of alignments selected from the CDD database. Although CDD is the most recent expert-curated database, there are other structure-based sequence alignment databases, e.g. HOMSTRAD[17] and FSSP[18]. It is possible that use of these other databases can alter the optimal values of these parameters. Also, adjustments may be indicated as the program is tested using more structure pairs and used more widely. However, we also expect that any adjustment will be small in magnitude and, in particular, SE will remain superior to a dynamic programming algorithm.

## Conclusion

SE algorithm produces more accurate sequence alignments from superimposed structures than the dynamic programming algorithms used in CHIMERA, LSQMAN or SHEBA, especially in pairs of proteins with low sequence or structure similarity. SE does not require gap penalties but the alignments have fewer gaps. SHEBA implemented with SE algorithm takes less CPU time and generates more accurate alignments than the original version with dynamic programming algorithm. It is available as a software package for implementing in other structure comparison programs.

## Methods

### Seed Extension algorithm

**Figure 7:**
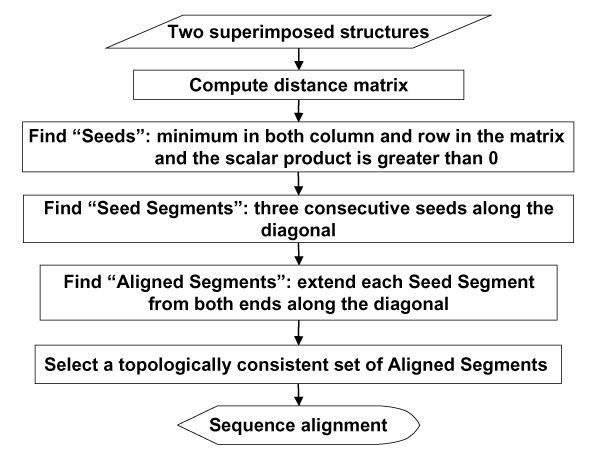
Overview of the Seed Extension algorithm.

The input of the algorithm is the coordinates of two superimposed structures and the output is the sequence alignment based on that superposition. The flow chart of the algorithm is shown in Figure 7.

#### 1. Compute distance matrix

Given a pair of superimposed structures A of length *m *and B of length *n*, the *m *× *n *matrix M of the average Cα distances is defined as

$$M_{i,j}=\sqrt{\frac{dist_{i-1,j-1}^{2}+dist_{i,j}^{2}+dist_{i+1,j+1}^{2}}{3}}$$

where *dist*_*i*, *j *_is the distance between the Cα atoms of residue *i *of structure A and residue *j *of structure B.

#### 2. Find seed and seed segments (SSs)

A pair of residues (*i*, *j*) is a seed if its corresponding matrix element *M*_*i*, *j *_is the minimum in both the *i*^*th *^row and the *j*^*th *^column and their scalar product is greater than 0. The scalar product here refers to that between unit vectors which bisect the angles (i-1, i, i+1) and (j-1, j, j+1). A seed segment (SS) is a set of consecutive seeds along one diagonal. After all seeds have been identified, seed segments of length 1 or 2 (isolated seeds or isolated pairs of seeds) are discarded and treated as not aligned. The status of each residue in structure A is stored as a "seed" or "extended pair" (see the following section), with the paired residue number in B, or "not yet aligned".

#### 3. Extend seed segments to obtain aligned segments (ASs)

**Figure 8:**
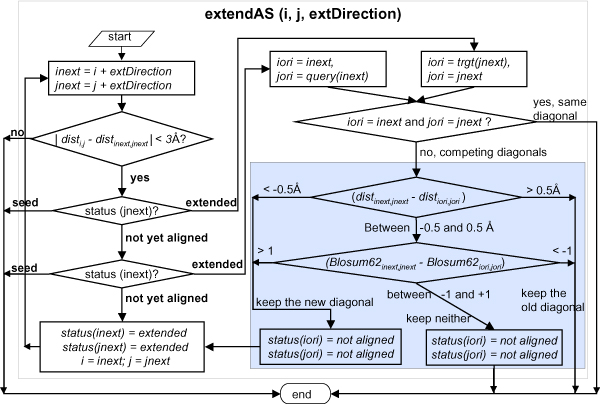
**Detailed flowchart of the seed extension process**. The terminal residue pair (*i*, *j*) of an aligned segment and the direction of the extension (extDirection) are the input. The value of extDirection is -1 or +1 for extension toward the N- or the C-terminus, respectively.

An aligned segment (AS) is a set of ungapped, at least 3 consecutive residue pairs that are aligned. ASs are initially set equal to SSs, which are then extended along the diagonal in both directions according to a protocol detailed in Figure 8. Briefly, an AS is extended by a residue pair if the distance between the new pair is not more than the distance between the last aligned pair by a cutoff distance (default is 3.0 Å). The extension is terminated if either of the candidate residue pair is a seed residue (a seed pair is preferred over an extended pair) or if the candidate residue pair is an extended pair (two ASs on the same diagonal are joined). If the extension meets a residue which is a part of a pre-existing AS on a different diagonal, the extension is either stopped or continued, in which case the pre-existing AS is correspondingly shortened, depending on which AS is to be preferred. The factors considered for this choice include the distance between the residue pairs and the similarity of the residue pairs as measured by the BLOSUM62 matrix.

#### 4. Collect consistent sets of diagonals and choose the best set

After all SSs are extended in both directions, a dynamic programming algorithm is used to choose the best set of consistent ASs. A set of ASs is consistent if for every AS pair p and q in the set, i_p _< i_q _implies j_p _< j_q_, where (i_p_, j_p_) and (i_q_, j_q_) are the starting residue numbers of the p^th ^and q^th ^ASs in the set, respectively. Consistency ensures that the resulting alignment respects sequence connectivity of the aligned residues in both structures. The best set of ASs was the one with the largest total number of aligned residue pairs in the set.

### SE, DP, and SHEBA modifications

The Seed Extension algorithm was first written as a standalone program called SE. In order to compare this algorithm with the dynamic programming algorithm, the dynamic programming routine in the program SHEBA was isolated into a standalone program, which we refer to as DP. Prior to the implementation of the Seed Extension algorithm into SHEBA, the program SHEBA was first modified by removing some known bugs and by altering some features of the refinement procedure. In SHEBA3.1, after initial alignment, the program enters seven different weight schemes of 3 superposition-alignment cycles each. The alignment that has the most number of aligned residues is chosen as the final alignment. This is the last updated version that still employs only the dynamic programming algorithm.

The new SHEBA4.2, with – dp option, also employs the dynamic programming algorithm but uses a modified iteration scheme. It also uses seven different weight schemes but, for each weight scheme, the program first runs three weighted superposition-alignment cycles followed by up to 10 unit weight cycles. If the alignment converged within 10 cycles, that is, the alignment did not change in two consecutive cycles, the converged alignment is selected; otherwise, the alignment which gives the most number of aligned residues in the next cycle is chosen for that weight scheme. The alignment that gives the largest number of aligned residues among the seven different weight schemes is chosen as the final alignment. SHEBA4.2 with – se option has the same iteration scheme but the dynamic programming algorithm in the alignment part of the superposition-alignment cycle is replaced by the Seed Extension algorithm.

### Data set of superimposed structures

A set of structurally aligned protein pairs was selected from NCBI's Conserved Domain Database as described below. In CDD version 2.09, there were 2009 expert curated families with names starting with 'cd', of which 593 had at least two protein sequences with PDB structure files available that did not contain missing coordinates or non-standard amino acid residues. From each of these families, the pair with the least sequence similarity was selected and structurally superimposed using CHIMERA [19] based on CDD alignments. Discarding those with Cα RMSD greater than 5 Å resulted in 582 protein pairs.

### Structure-based sequence alignment programs

We evaluated following programs for the accuracy of the sequence alignment generated from a given structural superposition: CHIMERA, LSQMAN version 060802, DP, and SE. The option GLocal_nw, global-superposition-distance-based Needleman-Wunsch sequence alignment, was used in LSQMAN to generate a sequence alignment from two superimposed structures. The default Cα distance cut-off value used in CHIMERA, LSQMAN and DP was 3.5 Å. Default values were used for the gap penalty.

### Alignment accuracy measure

The CDD alignments were used as reference alignments; those generated by programs were referred to as test alignments. The alignment accuracy was measured by means of the fraction of correctly aligned residues, *f*_*CAR*_. This is defined as the number of aligned pairs in the reference alignment that are preserved in the test alignment, divided by that in the reference alignment[20,21].

### Computing time measurement

To measure the speed of the algorithm, CPU time was retrieved by the *clock *function at the beginning and end of the DP or SE routine. The values were divided by the number of clock ticks per second to convert to the execution time. The average CPU time of SE or DP routine was obtained as the sum of elapsed time for all cycles divided by the number of cycles. The total CPU time to run the whole refinement cycles, including both the superposition and alignment generation, was also recorded. All time measurements were made on a Power Mac G5 with Dual PowerPC 970 2 GHz CPU.

## Competing interests

The authors declare that they have no competing interests.

## Authors' contributions

CHT implemented and improved the algorithm, performed the tests, and wrote the manuscript. JJV prepared the test set and wrote the routine that collects consistent set of diagonals. CK tested the programs. BL conceived the project, designed the algorithm and wrote the manuscript.
